# At What Age Does Strabismus Impact Quality of Life in Children? A Narrative Literature Review

**DOI:** 10.22599/bioj.489

**Published:** 2025-11-10

**Authors:** Aimee Cecil, Lauren Hepworth

**Affiliations:** 1 Cambridge University Hospitals NHS Foundation Trust, UK; 2 University College London, UK; 3 University of Liverpool, UK

**Keywords:** Strabismus, paediatric, quality of life, impact

## Abstract

**Background:**

Strabismus can have profound psychosocial impacts in addition to functional impacts. Psychosocial impacts typically stem from negative attitudes and can manifest as low self-esteem, employment discrimination, and problems forming interpersonal relationships. Evidence suggests that negative attitudes towards strabismus emerge in early childhood.

The aim of this review was to investigate the age at which strabismus impacts quality of life in children.

**Methods:**

A systematic search was conducted using online databases (MEDLINE, Embase, Emcare, and PsycINFO) from their inception until July 2024. The search strategy was derived from three topic areas: strabismus, quality of life, and children. A two-stage screening process involved screening titles and abstracts before full texts were retrieved and screened. Inclusion criteria required studies to have participants aged less than 18 years old with strabismus completing a measure of quality of life. Quality assessment was performed using the Strengthening the Reporting of Observational Studies in Epidemiology checklist.

**Results:**

From 1,014 records, a total of 10 studies were included. Most studies reported children with strabismus had significantly reduced quality of life compared to children without strabismus, across all age groups. Three studies compared scores between different age groups. One indicated a greater reduction in quality of life in older children. Conversely, two found no significant difference between different age groups.

**Discussion:**

Findings indicated that strabismic children across all age groups experience reduced quality of life, although age-specific analysis was limited. A longitudinal study using an appropriate, validated outcome measure from diagnosis until adulthood may facilitate an age-specific analysis of quality of life.

## Introduction

Strabismus describes a deviation from the normal orientation of one or both eyes, resulting in a misalignment of the visual axes so that both eyes cannot fixate on the same point simultaneously (Oystreck and Lyons, 2012). A meta-analysis by Hashemi *et al*. (2019) reported a global pooled prevalence of 1.9% for strabismus across all ages. Pathai, Cumberland and Rahi (2010) reported a prevalence of 2.1% among children in the United Kingdom (UK) specifically. Strabismus has a variety of functional impacts, such as diplopia, confusion, suppression, amblyopia, and loss of stereopsis (Buffenn, 2021). Pathological diplopia is usually experienced by adults and older children. Suppression is a sensory adaptation to prevent diplopia, involving mental inhibition of visual input from the deviating eye during binocular viewing, which typically develops in children up to 8 years old (Sharma *et al*., 2024). Treatment options for strabismus vary based on the classification and associated symptoms such as diplopia (McBain *et al*., 2014). Treatment options to manage the angle of the deviation include strabismus surgery, botulinum toxin injection, and refractive correction (Buffenn, 2021).

In the UK, the Royal College of Ophthalmologists (RCO) recognizes that botulinum toxin injection and surgery to correct strabismus are reconstructive, rather than cosmetic, procedures (RCO, 2016). Strabismus surgery to regain binocular single vision is universally regarded as plausible and functionally important (Durnian, Noonan and Marsh, 2011; Marsh, 2015). Contrastingly, surgery for asymptomatic strabismus with no potential for binocular single vision may be deemed less important by those who view it as a purely cosmetic procedure (Nelson *et al*., 2008). Since pediatric strabismus patients more commonly experience suppression, this belief is especially applicable for this population (Sharma *et al*., 2024). However, this belief disregards how strabismus can have significant psychosocial impacts as well as functional impacts (Marsh, 2015; Kushner, 2018).

Psychosocial impacts of strabismus include low self-esteem (Nelson *et al*., 2008), increased incidence of mental illness (Mohney *et al*., 2008), increased psychological distress, and increased interpersonal relationship difficulties due to misconceived negative attitudes from others and themselves (Al Shehri, Duan and Ratnapalan, 2020; Temeltürk, Koçer and Yaşar, 2022). Most psychosocial impacts of strabismus are related to issues with self-perception and perceptions from others (Buffenn, 2021). These issues likely stem from the significance placed on physical characteristics, such as body image and appearance, in society (Durnian, Noonan and Marsh, 2011). People from different cultures and age groups display similar facial attractiveness preferences, suggesting that desire for aesthetic qualities is an innate mechanism rather than a cultural influence (Cunningham *et al*., 1995; Slater *et al*., 2000; Ramsey *et al*., 2004; Little, Apicella and Marlowe, 2007). Visual perception of faces uses the nose, mouth, and eyes as the preferred targets for central gaze (Mertens, Siegmund and Grusser, 1993). The four specific qualities that underpin facial attractiveness are facial averageness, facial symmetry, youthfulness, and sexual dimorphism (Durnian, Noonan and Marsh, 2011). Evolutionary psychologists have connected these specific qualities to health, fitness, and developmental stability, which are key reproductive attributes (Zebrowitz and Montepare, 2008).

Human preference for facial symmetry is evident in the portrayal of characters in media. In animated films, characters with visible facial differences are often portrayed negatively (Sullivan *et al*., 2025). Characters with strabismus are significantly more likely to be portrayed as unintelligent, villainous, and followers rather than leaders (Liu *et al*., 2024). Due to the nature of animation, the animators have deliberately chosen to depict these characters in this way. As a result, children who watch these films may learn to associate visible facial differences, including strabismus, with negative characteristics. This exacerbates harmful stereotypes and social stigma (Liu *et al*., 2024; Sullivan *et al*., 2025).

With the importance of attractive appearance entrenched in humankind, it is unsurprising that individuals with visible differences can experience low self-esteem (Thornton *et al*., 2021). Nelson *et al*. (2008) investigated the psychosocial impacts of childhood-onset strabismus in 128 patients aged ≥15 years old who underwent reconstructive strabismus surgery. Through a 10-item questionnaire, over 80% of patients reported that their preoperative strabismus reduced their self-esteem, made them feel embarrassed, and caused difficulty making eye contact (Nelson *et al*., 2008). Furthermore, 85% of patients reported that reconstructive strabismus surgery increased their self-esteem (Nelson *et al*., 2008). These impacts can arise internally from negative self-perception or as a result of the behaviors and attitudes of others. Adults with strabismus can experience negative attitudes from others in the form of employment discrimination (Coats *et al*., 2000; Goff *et al*., 2006; Mojon-Azzi and Mojon, 2009) and may encounter prejudice when pursuing romantic relationships (Mojon-Azzi, Potnik and Mojon, 2008).

There is evidence that negative attitudes towards strabismus emerge in childhood. Paysse *et al*. (2001) observed children’s behavior towards strabismic dolls and reported that negative attitudes arose at approximately six years old. Three studies investigated children’s preferences for orthotropic versus strabismic peers using digitally manipulated photographs (Lukman *et al*., 2010; Lukman *et al*., 2011; Mojon-Azzi, Kunz and Mojon, 2011). Lukman *et al*. (2010) and Lukman *et al*. (2011) reported that children aged 5 to 6 years old and 8 to 12 years old, respectively, demonstrated significant preference for orthotropic children and negative attitudes towards peers with strabismus. Mojon-Azzi, Kunz and Mojon (2011) reported children aged <6 years old did not show any significant preference, while children aged ≥6 years old invited strabismic children to their birthday parties significantly less frequently than they invited orthotropic children when choosing one from a pair.

The literature demonstrates how negative attitudes towards strabismus prevail in many aspects of life, including throughout childhood. What is currently unknown is the time point at which these negative attitudes are recognized by children with strabismus and when this impacts their quality of life. Health-related quality of life refers to the subset of quality of life directly associated with an individual’s health, focusing on the emotional, functional, and social impacts of conditions (Carlton and Kaltenthaler, 2011). Health-related quality of life varies between patients with the same condition as well as within a patient over time, due to differences in experiences and expectations (Carr, Gibson and Robinson, 2001). Patient-reported outcome measures (PROMs) are standardized instruments that assess how individuals are impacted. These have been developed for both adult and pediatric populations and can be generic or condition-specific measures (Carlton and Kaltenthaler, 2011). These health-related quality of life measures allow the impact of strabismus to be assessed in a quantitative and standardized manner. The PROMs available specifically for use in pediatric populations, with proxy- and self-report versions, allow the childhood-specific impact of strabismus to be evaluated.

The aim of this review was to investigate the age at which strabismus impacts quality of life in children.

## Methods

This systematic literature review followed the Preferred Reporting Items for Systematic Reviews and Meta-Analyses (PRISMA) 2020 guidelines (Page *et al*., 2021). Study populations were required to be aged <18 years old and diagnosed with strabismus. Study designs including randomized controlled trials, controlled trials, cross-sectional studies, case-control studies, or cohort studies, either prospective or retrospective, were included. Letters, editorials, reviews, case series, and case reports were excluded. Studies were also required to use a PROM as an outcome measure for quality of life and include a comparison ‘normal’ population.

A systematic search was conducted using the following online databases: MEDLINE, Embase, Emcare, and PsycINFO, from inception until July 2024. The search strategy used subject heading terms and synonyms around the following topics: strabismus, quality of life, and children (Appendix 1).

Following de-duplication, a two-stage screening strategy was employed; EndNote was used to manage this process (Clarivate, 2023). Initially, titles and abstracts were screened by one reviewer. Subsequently, full texts were retrieved and assessed for eligibility by the same reviewer. Quality assessment of included studies was performed by one reviewer using the Strengthening the Reporting of Observational Studies in Epidemiology (STROBE) checklist (von Elm *et al*., 2007).

Data was extracted from the included studies by one reviewer and tabulated in pre-designed Microsoft Excel spreadsheets (Microsoft Corporation, 2021).

## Results

**Figure 1:**
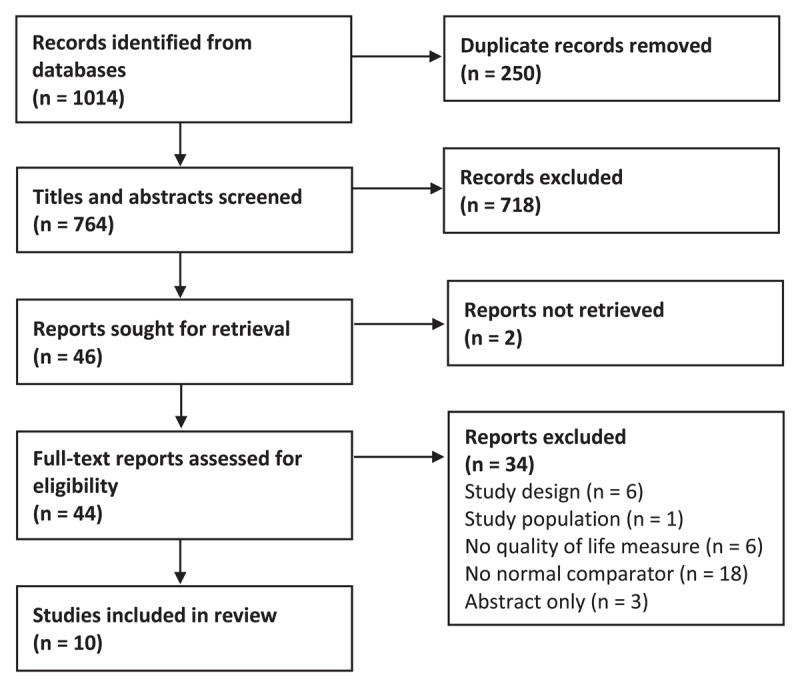
**Flow diagram outlining the review screening process**. Adapted from the PRISMA 2020 flow diagram template for systematic reviews (Page *et al*., 2021).

The search identified 1,014 records, of which 10 studies (20,446 participants, 2,178 with strabismus) met the inclusion criteria (Figure 1).

### Study characteristics

Five studies were cross-sectional studies with paired comparison groups (Sim, Yap and Chia, 2014; Tu *et al*., 2016; Hatt *et al*., 2020; Silva *et al*., 2022; Gouveia-Moraes *et al*., 2023), and two studies were cross-sectional studies with population-based controls (Wen *et al*., 2011; Schuster *et al*., 2019). The descriptions of paired comparison groups were heterogeneous. This included children with no strabismus recruited from a pediatric ophthalmology department (Gouveia-Moraes *et al*., 2023) children with no eye conditions and normal visual acuity (Tu *et al*., 2016; Silva *et al*., 2022) or children with no eye conditions, no refractive correction, and normal visual acuity (Hatt *et al*., 2020). Sim, Yap and Chia (2014) included two comparison groups: children with other eye conditions besides strabismus and amblyopia (group A) and children with no eye conditions (group B).

Two cross-sectional studies applied normal thresholds to PROM scores from strabismic children instead of comparing directly to controls (Hatt *et al*., 2022) or in addition to comparing directly to controls (Hatt, Leske and Holmes, 2010). Both studies classified individual PROM scores as either normal or below normal using 5^th^ percentile thresholds derived from normal controls. Hatt *et al*. (2022) defined their controls as children with no eye conditions, no refractive correction, and normal visual acuity, while Hatt, Leske and Holmes (2010) defined their controls as children with no strabismus and normal visual acuity.

One study included observational and interventional aspects. Chai *et al*. (2009) compared preoperative PROM scores with age- and gender-matched controls (exclusion criteria: chronic illness and visual impairment), as well as comparing quality of life scores before versus after reconstructive strabismus surgery. Only results from the former observational part of this study were relevant to the aim and therefore included in the review.

The sample sizes of each study varied greatly (Table 1), ranging from 36 children (Gouveia-Moraes *et al*., 2023) to 1,040 children with strabismus (Tu *et al*., 2016).

**Table 1:** Study characteristics.


STUDY	STUDY CHARACTERISTICS			

	STUDY DESIGN	SAMPLE SIZE (N)		COUNTRY

		STRABISMUS	CONTROLS	

Chai *et al*. (2009)	prospective interventional	60	60	China

Gouveia-Moraes *et al*. (2023)	cross-sectional	36	35	Portugal

Hatt, Leske and Holmes (2010)	cross-sectional	51	47	USA

Hatt *et al*. (2020)	cross-sectional	91	166	USA

Hatt *et al*. (2022)	cross-sectional	98	310	USA

Schuster *et al*. (2019)	cross-sectional	579	12,410	Germany

Silva *et al*. (2022)	cross-sectional	42	21	Portugal

Sim, Yap and Chia (2014)	cross-sectional	60	120	Singapore

Tu *et al*. (2016)	cross-sectional	1040	1002	China

Wen *et al*. (2011)	cross-sectional	121	4097	USA


### Quality assessment

The quality of the studies assessed using the STROBE checklist was variable (Appendix 2). Common areas of weakness across all studies were lack of detail in statistical methods and omitting an explanation of how the study size was calculated. When the title and abstract criteria were not fulfilled, this was due to studies failing to provide a commonly used term for the study design.

### Participant characteristics

The studies recruited participants within a variety of age ranges (Table 2). Four studies had age ranges spanning pre-school to high school (Chai *et al*., 2009; Schuster *et al*., 2019; Hatt *et al*., 2020; Silva *et al*., 2022), while three studies had age ranges spanning primary school to high school (Hatt, Leske and Holmes, 2010; Sim, Yap and Chia, 2014; Hatt *et al*., 2022). One study recruited solely pre-school-aged children (Wen *et al*., 2011), one study recruited solely primary school-aged children (Gouveia-Moraes *et al*., 2023), and one study recruited solely teenagers (Tu *et al*., 2016). The majority of studies were well balanced between males and females recruited (Table 2). Although ethnicity was poorly reported, the studies that did report ethnicity were either majority White (Hatt, Leske and Holmes, 2010; Hatt *et al*., 2020; Hatt *et al*., 2022), majority Chinese (Tu *et al*., 2016), or majority Hispanic and African American (Wen *et al*., 2011) (Table 2).

**Table 2:** Characteristics of participants in included studies. *NR = not recorded*.


REFERENCE	PARTICIPANT CHARACTERISTICS							

	AGE RANGE (YEARS)	SEX, MALE (%)	ETHNICITY (%)	TYPE OF STRABISMUS, n (%)	SIZE OF DEVIATION, n (%)	TIME SINCE STRABISMUS DIAGNOSIS (MONTHS)	TREATMENT RECEIVED, n (%)	AMBLYOPIA, n (%)

Chai *et al*. (2009)	0–15	46.7	NR	NR	NR	Phoria 64 ± 59; Tropia 63 ± 57	NR	NR

Gouveia-Moraes *et al*. (2023)	5–12	53.5	NR	Esotropia, 30 (83.3); Exotropia, 6 (16.7)	≤10^Δ^, 16 (44.4); 10–30^Δ^, 14 (38.9); >30^Δ^, 6 (16.7)	NR	Surgery, 26 (72.2)	6 (16.7)

Hatt, Leske and Holmes (2010)	5–16	37.3	White, 86	Intermittent exotropia, 51 (100)	NR	NR	Surgery, 1 (2); Glasses, 17 (33)	NR

Hatt *et al*. (2020)	0–17	54.9	White, 81.3; Asian, 4.4; American Indian, 2.2	Esotropia, 44 (48.4); Exotropia, 47 (51.6)	NR	NR	NR	NR

Hatt *et al*. (2022)	5–17	55.1	White, 73.5; Hispanic, 7.1; Asian, 4.1; American Indian, 4.1	Esotropia, 41 (41.8); Exotropia, 57 (58.1)	NR	NR	NR	34 (34.7)

Schuster *et al*. (2019)	3–17	51.1	NR	NR	NR	NR	NR	NR

Silva *et al*. (2022)	2–17	43.0	NR	Esotropia, 20 (48); Exotropia, 18 (43); Hypertropia 4 (9)	<10^Δ^, 17 (40); 10–39^Δ^, 20 (48); ≥40^Δ^, 5 (12)	39 (9–183)	Surgery, 24 (57); Prior occlusion, 27 (64); Occlusion, 8 (19); Glasses, 25 (60)	9 (21)

Sim, Yap and Chia (2014)	5–16	42.2	NR	Esotropia, 12 (20); Exotropia, 47 (80)	NR	NR	NR	NR

Tu *et al*. (2016)	Mean 15.6 (±1.2)	46.0	Chinese, 100	NR	NR	NR	NR	NR

Wen *et al*. (2011)	2–6	50.3	Hispanic, 50.5; African American, 49.5	NR	<10^Δ^, 11 (9.9); 10–30^Δ^, 79 (71.2); >30^Δ^, 21 (18.9)	NR	NR	71 (2.1)


In most studies, participants had either esotropia or exotropia (Table 2). Gouveia-Moraes *et al*. (2023) had the highest cases of esotropia (83.3%), whereas Sim, Yap and Chia (2014) had the highest cases of exotropia (80%). Chai *et al*. (2009) investigated both manifest and latent strabismus, and Hatt, Leske and Holmes (2010) investigated intermittent exotropia only.

The size of deviations, time since strabismus diagnosis, treatment received, and presence of amblyopia were poorly reported. In the study by Wen *et al*. (2011), most participants had deviations measuring 10^Δ^ to 30^Δ^ (71.2%), while few participants had deviations measuring <10^Δ^ (9.9%). By contrast, Silva *et al*. (2022) and Gouveia-Moraes *et al*. (2023) both had a greater proportion of participants whose deviations measured <10^Δ^ (40% and 44.4%, respectively) (Table 2). Time since strabismus diagnosis was reported as a mean of 63 months by Chai *et al*. (2009) and a median of 39 months by Silva *et al*. (2022). The majority of participants in the studies by Silva *et al*. (2022) and Gouveia-Moraes *et al*. (2023) had undergone previous reconstructive strabismus surgery (72.2% and 57%, respectively). In addition, Silva *et al*. (2022) reported that participants were treated with prior occlusion (64%), current occlusion (19%), and current glasses (60%). Only one participant (2%) had undergone previous reconstructive strabismus surgery in the study by Hatt, Leske and Holmes (2010), and one-third (33%) had current glasses. When reported, the presence of amblyopia varied greatly between studies, ranging from 2.1% (Wen *et al*., 2011) to 34.7% (Hatt *et al*., 2022).

### Patient Reported Outcome Measures

The studies used a range of PROMs, outlined in Table 3. Generic measures included the Pediatric Quality of Life Inventory (PedsQL) (Hatt, Leske and Holmes, 2010; Wen *et al*., 2011; Hatt *et al*., 2020; Gouveia-Moraes *et al*., 2023) and the Kinder Lebensqualität fragebogen (Children’s Quality of Life Questionnaire) (KINDL-R) (Schuster *et al*., 2019). Ophthalmic-specific measures included the Pediatric Eye Questionnaire (PedEyeQ) (Hatt *et al*., 2020; Hatt *et al*., 2022; Silva *et al*., 2022) and the National Eye Institute Visual Function Questionnaire (NEI VFQ25) (Chai *et al*., 2009; Tu *et al*., 2016). Strabismus-specific measures included the Intermittent Exotropia Questionnaire (IXTQ) (Hatt, Leske and Holmes, 2010; Sim, Yap and Chia, 2014) and the Adult Strabismus Questionnaire (AS-20) (Sim, Yap and Chia, 2014). For all these PROMs, a lower score indicates a lower quality of life.

**Table 3:** Patient reported outcome measure findings.


REFERENCE	PATIENT REPORTED OUTCOME MEASURE	SIGNIFICANT DIFFERENCE VS CONTROLS	SIGNIFICANT DIFFERENCE BETWEEN AGE GROUPS

Chai *et al*. (2009)	proxy-reported NEI VFQ-25	Yes (p < 0.05)	–

Gouveia-Moraes *et al*. (2023)	proxy-reported PedsQL	No	–

Hatt, Leske and Holmes (2010)	proxy-reported PedsQL	Yes (p = 0.04)	–

	self-reported PedsQL	No	–

	proxy-reported IXTQ	Yes (p = 0.04)	–

	self-reported IXTQ	Yes (p < 0.0001)	–

Hatt *et al*. (2020)	proxy-reported PedsQL	No	–

	self-reported PedsQL	No	–

	proxy-reported PedEyeQ	Yes (p < 0.01)	–

	self-reported PedEyeQ	Yes (p < 0.01)	–

Hatt *et al*. (2022)	proxy-reported PedEyeQ	-	No

	self-reported PedEyeQ	-	Yes (p < 0.04)

Schuster *et al*. (2019)	proxy-reported KINDL-R	Yes (p < 0.001)	–

	self-reported KINDL-R	Yes (p = 0.016)	–

Silva *et al*. (2022)	proxy-reported PedEyeQ	Yes (p < 0.04)	No

	self-reported PedEyeQ	Yes (p < 0.04)	No

Sim, Yap and Chia (2014)	proxy-reported IXTQ	No vs group A controls	No

		Yes vs group B controls (p < 0.001)	

	self-reported IXTQ	Yes vs group A controls (p = 0.001)	No

		Yes vs group B controls (p < 0.001)	

	proxy-reported AS-20	No vs group A controls	–

		Yes vs group B controls (p < 0.003)	

	self-reported AS-20	No vs group A controls	–

		Yes vs group B controls (p = 0.001)	

Tu *et al*. (2016)	self-reported NEI VFQ-25	Yes (p = 0.001)	–

Wen *et al*. (2011)	proxy-reported PedsQL	Yes (p < 0.05)	–


– = Not Investigated, AS-20 = Adult Strabismus Questionnaire, IXTQ = Intermittent Exotropia Questionnaire, KINDL-R = Kinder Lebensqualität fragebogen (Children’s Quality of Life Questionnaire), NEI VFQ-25 = 25-item National Eye Institute Visual Function Questionnaire, PedEyeQ = Pediatric Eye Questionnaires, PedsQL = Pediatric Quality of Life Inventory. Group A = children with other eye conditions besides strabismus and amblyopia. Group B = children with no eye conditions.

Three studies used parent-proxy reports only (Chai *et al*., 2009; Wen *et al*., 2011; Gouveia-Moraes *et al*., 2023), one study used self-reports only (Tu *et al*., 2016), and six studies used a combination of proxy- and self-reported measures (Hatt, Leske and Holmes, 2010; Sim, Yap and Chia, 2014; Schuster *et al*., 2019; Hatt *et al*., 2020; Hatt *et al*., 2022; Silva *et al*., 2022). Seven studies used a single PROM (Chai *et al*., 2009; Wen *et al*., 2011; Tu *et al*., 2016; Schuster *et al*., 2019; Hatt *et al*., 2022; Silva *et al*., 2022; Gouveia-Moraes *et al*., 2023), while three studies used multiple PROMs (Hatt, Leske and Holmes, 2010; Sim, Yap and Chia, 2014; Hatt *et al*., 2020) to assess quality of life.

The form of delivery used in the studies was typically interview or self-administered questionnaires. Three studies used exclusively in-person or telephone interviews (Chai *et al*., 2009; Wen *et al*., 2011; Gouveia-Moraes *et al*., 2023). Two studies used self-administered questionnaires for parent proxy reporters and older children self-reporting, while in-person interviews were used for younger children self-reporting (Hatt, Leske and Holmes, 2010; Hatt *et al*., 2022). Four studies used self-administered questionnaires for all participants, with assistance from a trained interviewer (Sim, Yap and Chia, 2014) or assistance from a member of the research team (Tu *et al*., 2016; Hatt *et al*., 2020; Silva *et al*., 2022) when required. One study did not disclose their form of delivery (Schuster *et al*., 2019).

Of the three studies that used multiple PROMs, the order of completing was not reported by two studies (Sim, Yap and Chia, 2014; Hatt *et al*., 2020), and one study had a standardized order of IXTQ followed by PedsQL (Hatt, Leske and Holmes, 2010).

### Quality of life compared to children without strabismus

Most studies reported that children with strabismus had significantly reduced quality of life compared to children without strabismus (Table 3). Chai *et al*. (2009) reported that strabismic children aged 0 to 15 years old scored significantly lower than controls in eight proxy-reported NEI VFQ25 subscales: ‘general health’, ‘general vision’, ‘near vision’, ‘distance vision’, ‘social functioning’, ‘mental health’, ‘dependency’, and ‘peripheral vision’. Tu *et al*. (2016) reported that teenagers with strabismus scored significantly lower than controls in 11 of 12 self-reported NEI VFQ-25 subscales, with no significant difference for the subscale ‘social functioning’. Similarly, Wen *et al*. (2011) reported that pre-school-aged children with strabismus scored significantly lower than controls in all proxy-reported PedsQL domains except ‘social functioning’. Silva *et al*. (2022) reported that strabismic children aged 2 to 17 years old scored significantly lower than controls in self-reported PedEyeQ domains ‘social’, ‘frustration/worry’, and ‘bothered by eyes/vision’, while there was no significant difference for the domain of ‘functional vision’. Using the proxy-reported PedEyeQ, strabismic children aged 2 to 17 years old scored significantly lower than controls in all domains (Silva *et al*., 2022). Schuster *et al*. (2019) reported that using proxy- and self-reports, total KINDL-R scores and the subscale ‘friends’ were significantly lower for strabismic children aged 3 to 17 years old than controls. Finally, Hatt *et al*. (2022) reported that the majority of children with strabismus aged 5 to 17 years old had below ‘normal’ proxy- and self-reported PedEyeQ scores.

In two studies that used multiple PROMs, scores for strabismic children were significantly lower than controls for at least one measure but not consistently across all (Table 3). Sim, Yap and Chia (2014) reported that with self-reported IXTQ, scores were significantly lower for children with strabismus compared to both controls with other eye conditions and controls without any eye conditions. In contrast, with the self-reported AS-20, there was no longer a significant difference compared to controls with other eye conditions (Sim, Yap and Chia, 2014). Hatt *et al*. (2020) reported that with proxy- and self-reported PedEyeQ, children with strabismus scored significantly lower than controls in all domains across all age groups. However, using the PedsQL, the only significant differences were for ‘physical functioning’ in children aged 13 to 17 years old (self-reported) and ‘school functioning’ in children aged 2 to 4 years old (proxy-reported) (Hatt *et al*., 2020).

One study reported most quality of life scores for strabismic children to be within the ‘normal’ range, despite having statistically significantly lower scores than children without strabismus. Hatt, Leske and Holmes (2010) reported that strabismic children aged 5 to 16 years old scored significantly lower than controls using the self-reported IXTQ, proxy-reported IXTQ, and proxy-reported PedsQL, although no significant difference was found using the self-reported PedsQL. When comparing to ‘normal’ thresholds, proxy-reported IXTQ was the single measure where the majority of strabismic children scored below ‘normal’ (55%). Only a small proportion of scores were below ‘normal’ when assessed using self-reported IXTQ (8%), self-reported PedsQL (14%), and proxy-reported PedsQL (18%) (Hatt, Leske and Holmes, 2010). This study was only one of two that applied ‘normal’ thresholds to quality of life scores obtained from strabismic children. This was achieved by classifying individual scores as either ‘normal’ or below ‘normal’ using 5^th^ percentile thresholds derived from controls. However, the control sample size was only n = 47; therefore, the ‘normal’ threshold was derived from a limited population and may explain why most scores for strabismic children were within the ‘normal’ range. By contrast, Hatt *et al*. (2022) derived their 5^th^ percentile thresholds from 310 controls and reported that the majority of strabismic children scored below ‘normal’. Also, in the study by Hatt, Leske and Holmes (2010), all participants had intermittent exotropia, and it is not surprising that due to the intermittent nature of the strabismus, they were less impacted (Na and Kim, 2016; Haggerty *et al*., 2004).

In contrast to the consensus, one study reported that children with strabismus did not have significantly lower quality of life scores compared to children without strabismus (Table 3). Gouveia-Moraes *et al*. (2023) reported that strabismic children aged 5 to 12 years old scored lower than controls in all proxy-reported PedsQL domains except ‘physical functioning’. However, there was no statistically significant difference in any domain nor in the total score. The lack of statistical significance may be attributable to the choice of controls, who were children recruited from a pediatric ophthalmology department. While no ocular misalignment was specified as a requirement for the controls, there was no exclusion of refractive correction, reduced visual acuity, or other eye conditions, which may also reduce quality of life (Gouveia-Moraes *et al*., 2023). This was illustrated by Sim, Yap and Chia (2014), who reported no significant difference in quality of life scores of strabismic children and controls with other eye conditions (assessed using proxy-reported IXTQ, proxy-reported AS-20, and self-reported AS-20). Contrastingly, there was a significant difference in quality of life scores for strabismic children compared to controls with no eye conditions. This highlights the necessity to have a clear distinction between populations.

Gouveia-Moraes *et al*. (2023) used a generic PROM: proxy-reported PedsQL, which is less sensitive to the specific impacts of eye conditions compared to condition-specific measures (McBain *et al*., 2014). This was also highlighted by Hatt *et al*. (2020), who reported no significant difference in scores between children with strabismus and controls when assessed using the PedsQL. However, Hatt *et al*. (2020) were able to demonstrate a statistically significantly reduced quality of life when assessed using an ophthalmic-specific measure: proxy-reported PedEyeQ. This is further highlighted by Hatt, Leske and Holmes (2010), who reported that the condition-specific measure, proxy-reported IXTQ, was more sensitive at detecting reduced quality of life in strabismic children compared to proxy-reported PedsQL. Items in PedsQL are not phrased in a way that relates to the eyes, nor do they explore specific issues related to ophthalmic conditions (Gouveia-Moraes *et al*., 2023).

A further reason that may explain the absence of statistical significance reported by Gouveia-Moraes *et al*. (2023) is the high proportion (44%) of children who had deviations measuring ≤10^Δ^, which may have resulted in fewer concerns regarding their strabismus. Furthermore, a high percentage (72.2%) of participants had previously undergone reconstructive strabismus surgery, which may have alleviated the impact (Gouveia-Moraes *et al*., 2023).

### Effects of age

Three studies included statistical analysis of quality of life scores between different age groups (Table 3). Hatt *et al*. (2022) reported that a significantly higher proportion of strabismic children aged 12 to 17 years old had below-‘normal’ self-reported PedEyeQ scores than those aged 5 to 11 years old, in all domains. The greatest difference between the two age groups was in the ‘social’ domain (89.3% versus 18.6%, respectively) (Hatt *et al*., 2022). Conversely, there was no significant difference between the two age groups in any domain when assessed using proxy-reported PedEyeQ (Hatt *et al*., 2022). Sim, Yap and Chia (2014) reported that strabismic children aged 5 to 7 years old had lower self-reported IXTQ scores than those aged 8 to 16 years old; however, this was not statistically significant. Silva *et al*. (2022) reported that there was no significant difference in proxy- and self-reported PedEyeQ scores between strabismic children aged 5 to 11 years old and 12 to 17 years old. Scores in self-reported PedEyeQ domains ‘social’, ‘functional vision’, and ‘bothered by eyes/vision’ were lower for younger strabismic children, while scores in the self-reported PedEyeQ ‘frustration/worry’ domain were lower for older strabismic children (Silva *et al*., 2022). The lack of statistical significance between age groups may be attributable to the small sample sizes in these studies, meaning they were insufficiently powered.

Two studies provided data for different age groups that allowed tentative comparisons to be made with no statistical analysis between age groups. Wen *et al*. (2011) reported that strabismic children aged 4 to 5 years old experienced problems more often across a larger number of proxy-reported PedsQL items than strabismic children aged 2 to 3 years old. In eight items (‘problems walking’, ‘running’, ‘participating in exercise’, ‘taking a bath or shower’, ‘experiencing aches’, ‘feeling sad’, ‘feeling angry’, and ‘not being able to do things that others can’), a significantly higher proportion of parents of strabismic children aged 4 to 5 years old selected ‘often’ or ‘always’ than parents of age-matched controls. By contrast, this only occurred in three items (‘trouble sleeping’, ‘worrying what will happen’, and ‘not being able to do things that others can’) for those aged 2 to 3 years old (Wen *et al*., 2011). In the study by Hatt *et al*. (2020), self-reported PedEyeQ scores appeared similar overall between strabismic children aged 5 to 11 years old and those aged 12 to 17 years old. Younger strabismic children had lower median scores in domains ‘functional vision’ and ‘bothered by eyes/vision’, while older strabismic children had lower median scores in domains ‘social’ and ‘frustration/worry’ (Hatt *et al*., 2020).

## Discussion

Strabismus can have detrimental psychosocial impacts on patients, which are now appreciated in addition to the well-recognized functional impacts of strabismus (Marsh, 2015). Negative attitudes towards the condition are present in many aspects of life, including employment, dating, and platonic friendships (Buffenn, 2021). This review aimed to investigate the age at which strabismus impacts quality of life for children. A total of 10 studies were reviewed. The findings indicate that strabismic children across all age groups have reduced quality of life; all except one study reported a statistically significant reduction in quality of life when measured using PROMs compared to controls. The variety of PROMs used and the different age groupings limited direct comparisons between studies. This impeded the ability to deduce an age at which quality of life is first impacted in strabismic children. When comparisons between different age groups were made within studies, the findings were heterogeneous, including older children being affected more and no differences reported between older and younger children.

One aspect that varied greatly among the studies included in our review was the use of proxy- or self-reports. While most studies used both, three studies used exclusively proxy-reported measures, and one study used a self-reported measure only. There is debate in the literature regarding the usefulness and accuracy of proxy reporting. Quality of life is a subjective measure that is dependent on the personal experiences of an individual, and self-reported measures are the principal assessment method (Hays *et al*., 1995). However, self-reporting by children may be unreliable if they do not yet have the required cognitive or language skills to understand questions and reflect on events long-term (Theunissen *et al*., 1998). Therefore, it may be useful to have parent proxy reporters who know the child sufficiently to give accurate information (Hays *et al*., 1995). Parent proxy reports can be a substitute perspective if children cannot complete a self-report or a complementary perspective providing additional insight to explore the quality of life of a child (Sherifali and Pinelli, 2007). Despite the benefits, parent proxy reports may have reduced accuracy. There can be differing views and standards between children and parents as well as varying closeness in parent-child relationships (Ronen *et al*., 2003). A systematic review by Upton, Lawford and Eiser (2008) reported that parents of children with health conditions were more inclined to underrate quality of life scores compared to their children. In addition, parents of children with no health conditions were more inclined to overrate quality of life scores compared to their children (Upton, Lawford and Eiser, 2008). This aligns with reports from studies in our review. Silva *et al*. (2022) reported that children with strabismus had significantly lower quality of life scores than controls in three PedEyeQ domains by self-report and in four domains by proxy-report. In addition, Hatt, Leske and Holmes (2010) reported that strabismic children had significantly lower quality of life scores than controls with proxy-reported PedsQL but not with self-reported PedsQL. There is the possibility that parents are projecting their own worries when completing proxy reports (Hatt, Leske and Holmes, 2010). It is recommended that parent proxy reports be used as a complementary perspective. This is to allow both child and parent views to assist with the interpretation of a child’s quality of life. In truth, substitute perspectives should only be used when children are too young or too unwell to self-report (Sherifali and Pinelli, 2007). Therefore, findings by Chai *et al*. (2009) and Gouveia-Moraes *et al*. (2023) are limited since they used substitute parent proxy reports despite recruiting capable participants up to 15 years old and 12 years old, respectively.

Another aspect that varied between studies was the criteria that defined controls. This ranged from children with other eye conditions, children with no eye conditions, with the exception of refractive correction, to children with no eye conditions and no refractive correction. This may have caused some of the heterogeneity in findings between studies. The importance of clearly describing the control population was illustrated by Sim, Yap and Chia (2014), who reported no significant difference in scores compared to controls with other eye conditions, while there was a significant difference in scores compared to controls with no eye conditions.

While most studies in our review directly compared average quality of life scores from strabismic children to average scores from controls, two studies applied ‘normal’ thresholds to individual scores from strabismic children. Hatt, Leske and Holmes (2010) demonstrated opposing findings with these two methods. Children with strabismus had a significantly reduced quality of life when average scores were compared to controls; however, most quality of life scores for strabismic children were within the ‘normal’ range (Hatt, Leske and Holmes, 2010). Utilizing normal thresholds in clinical practice may be beneficial to assess if children with strabismus are experiencing reduced quality of life and would facilitate a quick and convenient evaluation on an individual basis. However, to ensure accuracy, normal thresholds must be derived from a large sample of controls. Hatt *et al*. (2022) derived normal thresholds for PedEyeQ from 310 controls, reducing the confidence interval, and subsequently reported the majority of strabismic children scored below ‘normal’.

Ethnicity of participants in the studies was poorly reported, which concurs with findings from a systematic review by Kuo *et al*. (2024) investigating ethnic diversity in pediatric ophthalmology trials. When ethnicity was reported, Kuo *et al*. (2024) observed an overrepresentation of White individuals in US-based studies compared to the 2010 US census data. This was replicated in the current review, as White individuals were similarly overrepresented in the US-based studies by Hatt, Leske and Holmes (2010), Hatt *et al*. (2020), and Hatt *et al*. (2022) compared to the 2010 and 2020 US census data (US Census Bureau, 2020). It is important that participant demographics reflect the whole population to increase generalizability of results and overcome ethnic inequalities in healthcare (Kuo *et al*., 2024).

The limitations of our literature review include that the screening process was performed by one individual. However, the methodology followed PRISMA 2020 guidelines (Page *et al*., 2021) to ensure transparency and completeness of the review.

Although our literature review was unable to reveal a specific age at which strabismus impacts quality of life in childhood, the findings demonstrated that children across all ages experience reduced quality of life. It was anticipated that this literature review would identify a suitable age to recommend initiating treatment options, such as reconstructive strabismus surgery and counselling. However, this was not attainable. Nonetheless, the findings highlight how quality of life and mental health should be considered earnestly in all pediatric strabismus patients. Therefore, it may be beneficial to have a low threshold for referring these patients to mental health services or implementing a dedicated orthoptic department-based patient support service.

## Conclusion

In order to successfully deduce an age at which strabismus begins to impact quality of life for children, further research is required in the form of a longitudinal study. A validated age-appropriate PROM is recommended for this research. Future studies in this area should utilize input from psychologists, with a multidisciplinary approach having the potential to offer greater insight and more substantive results. In current RCO guidelines for the management of strabismus in children, there is no clear guidance on the ideal age for reconstructive strabismus surgery (RCO, 2012). If the age at which strabismus impacts quality of life for children can be determined, the findings could guide clinical decision-making regarding management of pediatric strabismus and would be useful for informing the optimal timing of reconstructive strabismus surgery.

## Additional File

The additional file for this article can be found as follows:

10.22599/bioj.489.s1 Appendix. Appendix 1–2.
