# Supplementary material for: At What Age Does Strabismus Impact Quality of Life in Children? A Narrative Literature Review
Source: Br Ir Orthopt J. 2025 Nov 10;21(1):105–17. doi: 10.22599/bioj.489 (PMC12617420; doi:10.22599/bioj.489)
Supplement: Appendix. — Appendix 1–2. [file bioj-21-1-489-s1.pdf]

Appendix 1 – Example search strategy

**Example search strategy.** *exp = explode (includes selected subject heading and all narrower terms below the heading). mp = multi-purpose (keyword search). / = medical subject heading search. * = any group of characters, including no character. ? = zero or one character.*

| **Search line** | **Search term** |
|---|---|
| 1 | exp Strabismus/ |
| 2 | strabismus.mp. |
| 3 | esotrop*.mp. |
| 4 | exotrop*.mp. |
| 5 | squint.mp. |
| 6 | 1 OR 2 OR 3 OR 4 OR 5 |
| 7 | exp "Quality of Life"/ |
| 8 | quality of life.mp. |
| 9 | exp Psychology, Social/ |
| 10 | Psychosocial Functioning/ |
| 11 | psychosocial impact.mp. |
| 12 | 7 OR 8 OR 9 OR 10 OR 11 |
| 13 | Pediatrics/ |
| 14 | p?ediatric*.mp. |
| 15 | exp Child/ |
| 16 | child*.mp. |
| 17 | Adolescent/ |
| 18 | adolescent.mp. |
| 19 | teenag*.mp. |
| 20 | 13 OR 14 OR 15 OR 16 OR 17 OR 18 OR 19 |
| 21 | 6 AND 12 AND 20 |

Appendix 2 – Quality Assessment

**Quality assessment of included studies using STROBE checklist** (von Elm *et al.*, 2007). *1 = title and abstract. 2 = background. 3 = rationale. 4 = study design. 5 = setting. 6 = participants selection. 7 = variables. 8 = data measurement. 9 = bias. 10 = study size. 11 = quantitative variables. 12 = statistical methods. 13 = participant results. 14 = descriptive data. 15 = outcome data. 16 = main results. 17 = other analyses. 18 = key results summarised. 19 = limitations. 20 = interpretation. 21 = generalisability. 22 = funding.*

☑ = fulfilled. ☒ = not fulfilled. ? = insufficient information.

| **Study** | **Title/ abstract** | **Introduction** | | **Methods** | | | | | | | | | **Results** | | | | | **Discussion** | | | | **Other** | **Total, n (%)** |
|---|---|---|---|---|---|---|---|---|---|---|---|---|---|---|---|---|---|---|---|---|---|---|---|
| | **1** | **2** | **3** | **4** | **5** | **6** | **7** | **8** | **9** | **10** | **11** | **12** | **13** | **14** | **15** | **16** | **17** | **18** | **19** | **20** | **21** | **22** | |
| Chai *et al.* (2009) | ☒ | ☑ | ☑ | ☑ | ☑ | ☒ | ? | ☑ | ☒ | ☒ | ☑ | ? | ☑ | ☑ | ☑ | ☒ | ? | ☑ | ☒ | ☑ | ☒ | ☒ | 11 (50) |
| Gouveia-Moraes *et al.* (2023) | ☑ | ☑ | ☑ | ☑ | ☑ | ☒ | ? | ☑ | ☒ | ☒ | ☑ | ? | ? | ☑ | ☑ | ☒ | ? | ☑ | ☒ | ☑ | ☒ | ☑ | 12 (55) |
| Hatt, Leske and Holmes (2010b) | ☒ | ☑ | ☑ | ☑ | ☒ | ☒ | ? | ☑ | ☒ | ☒ | ☑ | ? | ? | ☑ | ☑ | ☒ | ☒ | ☑ | ☑ | ☑ | ☒ | ☒ | 10 (45) |
| Hatt *et al.* (2020) | ☑ | ☑ | ☑ | ☑ | ☑ | ☒ | ☑ | ☑ | ☒ | ☒ | ☑ | ? | ? | ☑ | ☑ | ☑ | ? | ☑ | ☑ | ☑ | ☑ | ☑ | 16 (73) |
| Hatt *et al.* (2022) | ☒ | ☑ | ☑ | ☑ | ☒ | ☒ | ? | ☑ | ☒ | ☒ | ☑ | ? | ? | ☑ | ☑ | ☑ | ? | ☑ | ☑ | ☑ | ☑ | ☑ | 13 (59) |

| Study | Title/ abstract | Introduction | | Methods | | | | | | | | | Results | | | | | Discussion | | | | Other | Total, n (%) |
|---|---|---|---|---|---|---|---|---|---|---|---|---|---|---|---|---|---|---|---|---|---|---|---|
| | 1 | 2 | 3 | 4 | 5 | 6 | 7 | 8 | 9 | 10 | 11 | 12 | 13 | 14 | 15 | 16 | 17 | 18 | 19 | 20 | 21 | 22 | |
| Schuster *et al.* (2019) | ☑ | ☑ | ☑ | ☑ | ☑ | ☑ | ☑ | ☑ | ☒ | ☒ | ☒ | ? | ☑ | ☑ | ☑ | ☑ | ? | ☑ | ☑ | ☑ | ☒ | ☑ | 16 (73) |
| Silva *et al.* (2022) | ☑ | ☑ | ☑ | ☑ | ☑ | ☒ | ? | ☑ | ☒ | ☒ | ☑ | ? | ? | ☑ | ☑ | ☑ | ? | ☑ | ☑ | ☑ | ☑ | ☑ | 15 (68) |
| Sim, Yap and Chia (2014) | ☒ | ☑ | ☑ | ☑ | ☒ | ☒ | ? | ☑ | ☒ | ☒ | ☑ | ? | ? | ☑ | ☑ | ☒ | ? | ☑ | ☑ | ☑ | ☒ | ☒ | 10 (45) |
| Tu *et al.* (2016) | ☒ | ☑ | ☑ | ☑ | ☒ | ☒ | ? | ☑ | ☒ | ☑ | ☑ | ? | ? | ☑ | ☑ | ☒ | ? | ☑ | ☑ | ☑ | ☑ | ☒ | 12 (55) |
| Wen *et al.* (2011) | ☑ | ☑ | ☑ | ☑ | ☒ | ☑ | ☑ | ☑ | ☒ | ☑ | ☑ | ? | ☑ | ☑ | ☑ | ☒ | ? | ☑ | ☒ | ☑ | ☑ | ☒ | 15 (68) |
